# Effect of Proinflammatory Cytokines (IL-6, TNF-***α***, and IL-1***β***) on Clinical Manifestations in Indian SLE Patients

**DOI:** 10.1155/2014/385297

**Published:** 2014-12-07

**Authors:** Vinod Umare, Vandana Pradhan, Milind Nadkar, Anjali Rajadhyaksha, Manisha Patwardhan, Kanjaksha K. Ghosh, Anita H. Nadkarni

**Affiliations:** ^1^National Institute of Immunohaematology, Indian Council of Medical Research, Mumbai, Maharashtra 400012, India; ^2^Department of Rheumatology, King Edward Memorial Hospital, Parel, Mumbai, Maharashtra 400012, India; ^3^National Institute of Immunohaematology, King Edward Memorial Hospital, 13th Floor, NMS Building, Parel, Mumbai 400012, India

## Abstract

Systemic lupus erythematosus (SLE) is an inflammatory rheumatic disease characterized by production of autoantibodies and organ damage. Elevated levels of cytokines have been reported in SLE patients. In this study we have investigated the effect of proinflammatory cytokines (IL-6, TNF-*α*, and IL-1*β*) on clinical manifestations in 145 Indian SLE patients. One hundred and forty-five healthy controls of the same ethnicity served as a control group. Clinical disease activity was scored according to SLEDAI score. Accordingly, 110 patients had active disease and 35 patients had inactive disease. Mean levels of IL-6, TNF-*α*, and IL-1*β* were found to be significantly higher in SLE patients than healthy controls (*P* < 0.001). Mean level of IL-6 for patients with active disease (70.45±68.32 pg/mL) was significantly higher (*P* = 0.0430) than those of inactive disease patients (43.85±63.36 pg/mL). Mean level of TNF-*α* was 44.76±68.32 pg/mL for patients with active disease while it was 25.97±22.03 pg/mL for those with inactive disease and this difference was statistically significant (*P* = 0.0161). Similar results were obtained for IL-1*β* (*P* = 0.0002). Correlation between IL-6, TNF-*α*, and IL-1*β* serum levels and SLEDAI score was observed (*r* = 0.20, *r* = 0.27, and *r* = 0.38, resp.). This study supports the role of these proinflammatory cytokines as inflammatory mediators in active stage of disease.

## 1. Introduction

Systemic lupus erythematosus (SLE) is a prototypic autoimmune disease characterized by the lack of tolerance to self-tissues and production of autoantibodies against a wide range of self-antigens like histones, DNA, RNA, ribosomal proteins, and other nuclear components [1, 2]. The imbalance in production of inflammatory cytokines like Interleukin-6 (IL-6), tumor necrosis factor-*α* (TNF-*α*), Interleukin-1 (IL-1), type I and type II interferons and Interleukin-10 (IL-10) contributes to immune dysfunction and also mediates inflammation of the tissues and organ damage [3, 4]. Several studies have shown that B-cell and T-cell hyperactivity and autoantibody production were associated with elevated levels of these proinflammatory cytokines [5, 6]. Some preliminary studies showed conflicting results of serum concentration of some inflammatory cytokines and disease activity in SLE patients [7, 8].

Il-6 is a pleiotropic cytokine produced in response to inflammatory stimuli. It is a key regulator of various cellular processes comprising erythropoiesis, bone metabolism, and neuronal cell degeneration. The role of IL-6 is controversial. Several studies on experimental models of SLE had shown an association of IL-6 with progression of lupus nephritis [9, 10]. TNF-*α* exhibits both the proinflammatory and immunoregulatory properties of cytokines. It appears to play an immunoregulatory role in differentiation of B-cells, T-cells, and dendritic cells. It also helps to execute the process of programmed cell death. Preliminary studies on lupus prone mice models have documented high concentrations of TNF-*α* in both sera and renal tissue and they were correlated with severity of kidney disease [11, 12]. Several studies have shown correlation of overexpression of TNF-*α* with disease activity and production of anti-dsDNA antibodies in SLE patients [13, 14]. Interleukin-1*β* (IL-1*β*), a member of IL-1 cytokine family, is a pleiotropic and immunoregulatory cytokine [15]. IL-1 family consists of two major agonistic proteins called IL-1*α* and IL-1*β* and one IL-1 receptor antagonist [16]. Overproduction of IL-1*β* has been documented to be involved in the pathogenesis of SLE and other autoimmune diseases [17–19].

In view of insufficient data about role of proinflammatory cytokines in the Indian SLE patients, this study was conducted to assess the role of TNF-*α*, IL-6, and IL-1*β* in clinical disease activity in SLE patients.

## 2. Materials and Methods

One hundred and forty-five (134 female and 11 male) patients fulfilling the American College of Rheumatology (ACR) classification criteria for SLE and without any concurrent infections were recruited in the study [20]. One hundred and forty-five age and sex matched healthy individuals were included as controls of the same ethnic background. The patients and healthy individuals with pregnancy, malignancies, iron deficiency anemia (IDA), and age more than 55 years were excluded from the study. The study was approved by institutional ethics committee (IEC). The written consent was taken from all patients and controls. The mean age of SLE patients at the time of evaluation was 28 ± 10 years. The mean disease duration was 2.6 ± 2.3 years. Severity of the disease was assessed by calculating SLE Disease Activity Index (SLEDAI) [8, 21]. The mean ± SD of SLEDAI was found to be 16.80 ± 7.62 among patients. Based on the SLEDAI score, patients were categorized into two groups, namely, active (SLEDAI ≥ 11) and inactive (SLEDAI ≤ 11). Accordingly there were 110 (100 females and 10 males) patients in active group and 35 (34 female and one male) patients in inactive group.

For urinalysis twenty-four-hour urine was collected. The peripheral blood was collected in plain bulb for the estimation of serum cytokines, creatinine, albumin, cholesterol, bilirubin, and calcium levels and for virological analyses (HBs Ag, HIV, and HCV antibodies). Anti-nuclear anti-bodies (ANA) were detected by using indirect immunofluorescence (IIF) method and serum complement components (C3; C4) levels were measured by Nephelometer (BN Prospec, Germany). The blood collected in EDTA was used for hematological analysis and erythrocyte sedimentation rate (ESR1 and ESR2). The cytokine levels were detected by bead based MILLIPLEX* MAP* technology (Millipore Corporation, Billerica, MA, USA). The limit of detection of cytokines was <3.5 pg/mL. Samples were run in duplicate and the calibrated recombinant protein was used to generate a standard curve.

### 2.1. Statistical Analysis

Mean ± standard deviation (SD) value was calculated for continuous variables and proportions for categorical variables. Means between two groups were analyzed by using unpaired Student's *t*-test. Fischer's exact (from Graph Pad online website) test was used to determine the association between laboratory investigations, clinical manifestations, and production of autoantibodies in SLE patients. Pearson correlation test was used to analyze the correlations between various laboratory measures and SLEDAI scores. A *P* value ≤0.05 was considered statistically significant.

## 3. Results

The cases and healthy individuals were matched regarding age and sex. No statistically significant association was observed between active and inactive patients regarding age, sex, and disease duration. The serum creatinine levels and 24-hour urine proteins levels were significantly higher in active SLE as compared to inactive group patients (*P* < 0.001). Complement levels and ESR after first and second hour were comparable in both the groups (Table 1).

**Figure 1:**
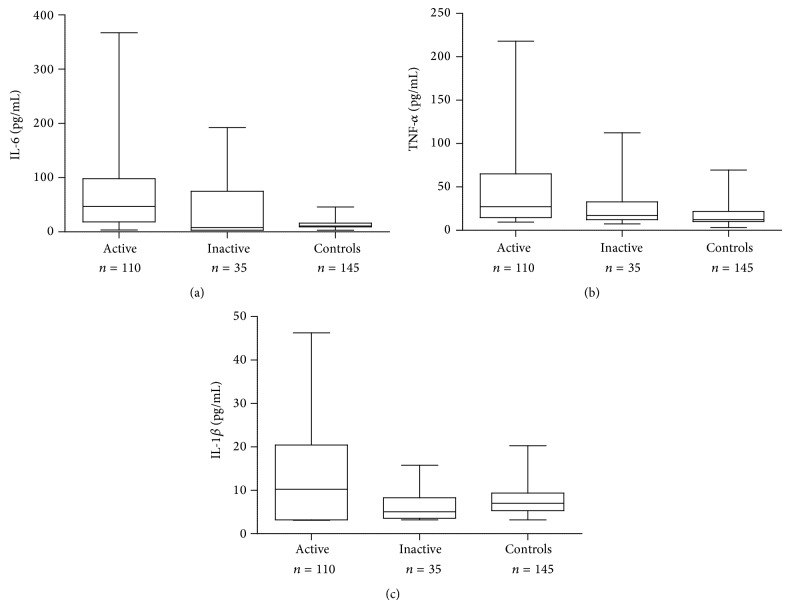
Distribution of serum cytokine levels in SLE patients with active and inactive disease along with healthy controls.

Serum levels of cytokines IL-6, TNF-*α*, and IL-1*β* were measured in all 145 SLE patients and 145 healthy individuals by cytokine multiplex assay.  Figure 1 shows the distribution of serum cytokine levels among patients and control groups. The mean level of IL-6 in SLE patients was 4.5 times higher (63.00 ± 67.28 pg/mL) as compared to controls (14.13 ± 8.61 pg/mL). This difference was statistically significant as compared with healthy individuals (*P* < 0.0001). The serum IL-6 level was significantly higher in active SLE patients (70.45 ± 68.32 pg/mL) as compared to inactive disease (43.83 ± 63.36 pg/mL, *P* = 0.0430) (Figure 1(a)).

The mean serum level of TNF-*α* also was significantly higher among SLE patients (40.17 ± 40.46 pg/mL) as compared to healthy individuals (17.35 ± 9.32 pg/mL, *P* < 0.0001). TNF-*α* levels were found to be significantly elevated (44.76 ± 68.32 pg/mL) in active SLE as compared to inactive group (25.97 ± 22.03 pg/mL, *P* = 0.0161) (Figure 1(b)). Serum levels of IL-1*β* was found to be significantly higher among SLE patients (11.48 ± 9.97 pg/mL) as compared with control group (7.89 ± 3.65 pg/mL, *P* = 0.0017). The mean serum level of IL-1*β* was significantly higher among SLE patients with active disease (13.21 ± 10.76 pg/mL) as compared to those with inactive disease (6.23 ± 3.27 pg/mL, *P* = 0.0002) (Figure 1(c)).

### 3.1. Association of Serum Cytokine Levels with Clinical Manifestations

It was observed that, out of total SLE patients studied, 57.27% patients in active SLE group were having renal disorders as compared to 20% of them in inactive group. The association was found to be statistically significant (*P* = 0.0002). Moreover, 12.72% patients with active disease were found to have involvement whereas none of the patients from inactive SLE group had neurologic disorder, Table 2. The clinical manifestations were then compared for the association with the serum levels of IL-6, TNF-*α*, and IL-1*β* among patients. The mean serum level of IL-6 in SLE patients with renal involvement in active disease was found to be increased (76.97 ± 54.62 pg/mL) when compared with inactive disease (23.93 ± 12.59). The association was found to be statistically significant (*P* = 0.013).

The serum TNF-*α* level in active SLE patients with renal involvement was observed to be significantly elevated (49.39 ± 45.46 pg/mL) as compared with patients in inactive disease group (13.53 ± 2.26 pg/mL, *P* = 0.0420). Similarly, mean serum level of IL-1*β* was also found to be significantly higher in active disease patients with renal involvement as compared with the patients with inactive disease (16.60 ± 11.28 pg/mL versus 6.40 ± 3.08 pg/mL, *P* = 0.0207). Although none of the patients with inactive disease showed neurologic disorder, their counterparts showed higher mean levels of serum TNF-*α* and IL-1*β* (41.50 ± 33.98 pg/mL and 20.53 ± 16.00 pg/mL, resp.).

### 3.2. Correlation of SLEDAI with IL-6, TNF-*α*, and IL-1*β*

**Figure 2:**
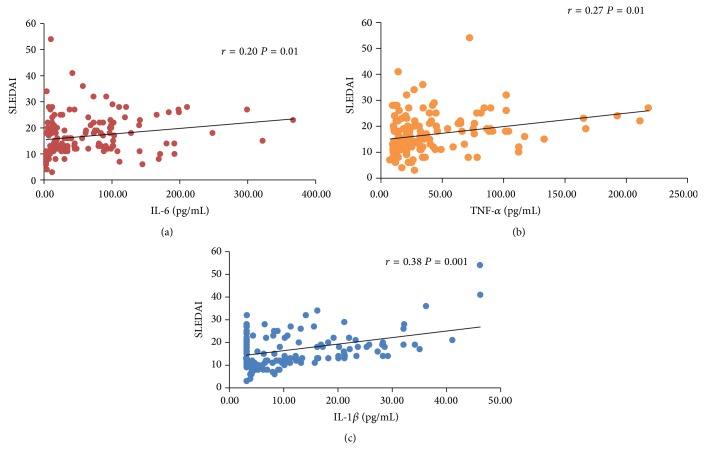
Correlation analysis of IL-6, TNF-*α*, and IL-1*β* with SLEDAI.

Figure 2 shows the correlation analysis of cytokine levels and SLEDAI scores. All the three cytokines showed a positive correlation with SLEDAI score (IL-6 *r* = 0.20; TNF-*α* *r* = 0.27; IL-1*β* *r* = 0.38). The patients from active group also showed the correlation between cytokine levels (IL-6 *r* = 0.10; TNF-*α* *r* = 0.20; IL-1*β* *r* = 0.25). In active renal disease patients, the IL-1*β* levels were positively correlated with SLEDAI score (*r* = 0.33, *P* < 0.001) compared with TNF-*α* (*r* = 0.18, *P* = 0.001) and IL-6 (*r* = 0.11, *P* = 0.01). Moreover, SLE patients in active disease having neurologic disorders showed higher cytokine levels. Although the IL-1*β* levels were found to be the highest among patients with neurologic manifestations (*r* = 0.50), the correlation was not statistically significant (*P* > 0.05).

**Figure 3:**
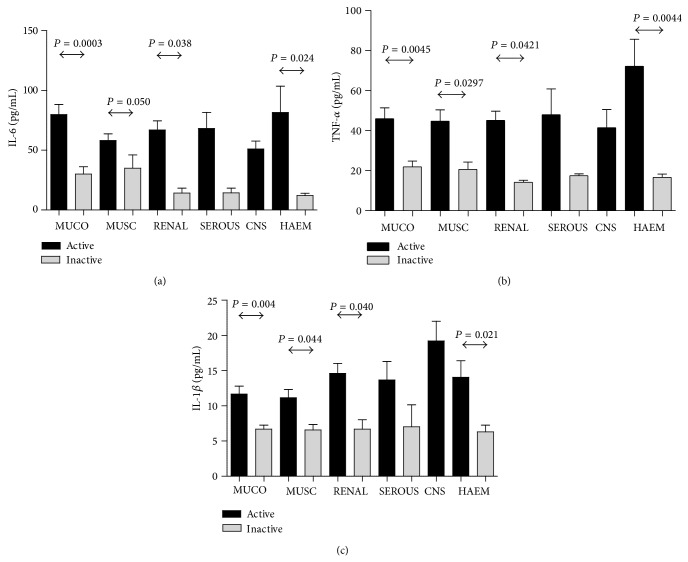
Bar chart showing levels of (a) IL-6, (b) TNF *α*, and (c) IL1*β* in active and inactive SLE patients with involvement of various organ systems. MUCO = mucocutaneous (malar/discoid rash, alopecia, oral ulcers, and photosensitivity), MUSC = musculoskeletal (myositis, arthritis, and arthralgia), RENAL = (urinary casts, hematuria >0.5 gm/day, proteinuria > 5 red blood cells/high-power field), SEROUS = serositis (pleurisy, pericarditis, and inflammation of peritoneum), CNS = central nervous system (seizure and neuropsychosis), HAEM = haematological (leukocytopenia, thrombocytopenia, and low haemoglobin concentrations), and *P* = statistical *P* value.

Figure 3 shows the distribution of serum IL-6, TNF-*α*, and IL-1*β* levels in clinical subsets such as mucocutaneous, musculoskeletal, renal, serous, CNS, and hematological subsets among SLE patients. The significantly high levels of IL-6, TNF-*α*, and IL-1*β* were observed in active musculoskeletal, renal, and hematological subsets as compared to patients with inactive subsets (*P* < 0.05).

To see if the expression pattern of the proinflammatory cytokines (IL-6, TNF-*α*, and IL-1*β*) in Indian SLE patients differs from other ethnic groups, we tried to correlate our results with results from other studies (Table 3). We observed that the expression of the proinflammatory cytokines in our population is not different than that of the other ethnic groups; however, it is more comparable with Caucasian, Brazilian, and Korean ethnicities.

## 4. Discussion

Systemic lupus erythematosus (SLE) is characterized by autoantibodies and mediated by formation of immune complexes. The imbalance between pro- and anti-inflammatory cytokines is a hallmark of the pathogenesis of SLE. Role of proinflammatory cytokines in pathogenesis of SLE is controversial. It has been demonstrated that proinflammatory cytokines such as IL-10, IL-6, TNF-*α*, and IL-1*β* show altered levels in SLE patients. Several studies have investigated various cytokine profiles among SLE patients in vitro and in vivo. Sabry et al. had reported the high serum levels of TNF-*α* and IL-6 in Egyptian SLE patients with active disease [7], while a study of AI-Janadi et al. had reported increased levels of these cytokines only in minority patients having active disease along with thrombocytopenia [6].

In our SLE patient cohort, all the studied cytokines were significantly elevated as compared to healthy controls (*P* < 0.05) which was similar to other studies [8, 23, 27]. The serum concentrations of these proinflammatory cytokines were found significantly higher in SLE patients with the active disease as compared with the patients with inactive disease (*P* < 0.05) implicating its active role in development of clinical presentation and disease severity. An association of raised IL-6 levels with lupus nephritis has been reported earlier [28, 29]. Ripley et al. in their study reported the raised levels of IL-6 and showed its correlation with the anemia in SLE patients of different ethnic origins [25]. We noted a significant increase in IL-6 levels among active SLE patients (70.45 ± 68.32 pg/mL) than that of inactive SLE (43.83 ± 63.36 pg/mL) as well as healthy controls. Higher levels of IL-6 were noted mainly in SLE patients with active renal disorder (*P* = 0.013). Several studies on experimental models of SLE had shown an association of IL-6 with progression of lupus nephritis [28, 30]. Herrera-Esparza et al. had demonstrated an increased in situ expression of IL-6 in lupus nephritis [31]. Jara et al. 1998 had reported an increased levels of CSF and serum IL-6 levels in SLE with CNS involvement [32]. We also found the increased levels of serum IL-6 in active patients with neurologic involvement. The IL-6 levels in active NPSLE were significantly raised as compared to inactive SLE patients and healthy controls (*P* < 0.05). The correlation of IL-6 levels and SLEDAI score showed conflicting results. We observed a positive correlation between IL-6 levels and SLEDAI (*r* = 0.20, *P* = 0.001). The same results were reported by Sabry et al. and Chun et al. [7, 24]. On the contrary, Gröndal et al. reported no correlation between IL-6 levels and overall disease activity either by SLEDAI or by SLAM (SLE activity measure) [26].

Several studies have shown the correlation of the overexpression of TNF-*α* with disease activity and production of anti-dsDNA antibodies in SLE patients [13, 14]. TNF-*α* mediates inflammation and renal tissue destruction in lupus nephritis patients [33]. In our study, the mean serum levels of TNF-*α* were found to be significantly increased in SLE patients compared to healthy controls (*P* < 0.0001). Similar findings were reported by Farid et al. [28] and Weckerle et al. [14]. Sabry et al. had reported significantly high TNF-*α* levels in active renal compared to inactive renal disease patients (*P* = 0.0420) among SLE patients from Egypt [7]. Similar findings were reported by Esposito et al. among Italian SLE patients where a positive correlation (*r* = 0.27, *P* = 0.0001) between the disease activity (SLEDAI) and the TNF-*α* levels was reported [30]. A study of Rana et al. in pediatric SLE patients from North India had reported an overexpression of TNF-*α* among 90% of patients where there was no correlation found between the levels of TNF-*α* with active neurologic disorders [8]. In accordance with a study by Aringer and Smolen [19], we also have found an association of increased levels of IL-1*β* with renal involvement among SLE patients. Table 3 shows the comparative analysis of the proinflammatory cytokines (IL-6, TNF-*α*, and IL-1*β*) among SLE patients and healthy controls from different ethnic groups and our SLE cohort studied. We observed that the expression of the proinflammatory cytokines in Indian population is not different than that of the other ethnic groups; however, it is more comparable with Caucasian, Brazilian, and Korean ethnicities.

Though the complement component (C3 and C4) levels are believed to be inversely proportional to the disease severity, reported results are inconsistent. In our SLE cohort, the serum levels of C3 were found to be significantly reduced in active SLE as compared to inactive one (*P* < 0.0114). Though C4 levels were reduced in active group, it did not achieve a significant difference when compared with inactive patients (Table 1). Similar results were reported by Rezaieyazdi et al. in their study in Iranian SLE patients. They found no significant difference in the complement levels among their active and inactive SLE patients [34].

This study suggests that proinflammatory cytokine milieu is altered among Indian SLE patients. This is reflected in an active stage of disease by a significant correlation between raised cytokines and SLEDAI. Clinical manifestations of renal and neurological involvement in active SLE patients further support the role of these proinflammatory cytokines as inflammatory mediators in active stage of disease.

## Figures and Tables

**Table 1:** Laboratory investigations for SLE patients.

Parameter	Active (*n* = 110)	Inactive (*n* = 35)	*P* value
24-hour urine proteins (g)	3.87 ± 1.14	1.83 ± 0.11	**0.0001**
Serum creatinine (mg/dL)	1.88 ± 1.49	1.28 ± 0.23	**0.0192**
Serum albumin (g/mL)	1.69 ± 0.84	2.01 ± 0.69	**0.0428**
ESR 1 (mm/h)	82.71 ± 48.21	65.81 ± 34.03	0.068
ESR 2 (mm/h)	98.34 ± 31.05	86.28 ± 49.11	0.087
Hemoglobin (g/dL)	9.38 ± 2.27	10.2 ± 2.04	0.058
Complement C3 (mg/dL)	73.72 ± 42.07	95.52 ± 49.02	**0.0114**
Complement C4 (mg/dL)	14.03 ± 8.95	15.17 ± 9.04	0.5137

**Table 2:** The occurrence of clinical manifestations of the disease in active (*n* = 110) and inactive (*n* = 35) SLE patients.

Clinical manifestations	Active (*n* = 110)	Inactive (*n* = 35)	*P* value
	*n* (%)	*n* (%)	
Rash (malar/discoid)	42 (38.18)	16 (45.71)	0.4360
Photosensitivity	39 (35.49)	07 (20)	0.0988
Arthritis	68 (61.81)	19 (54.29)	0.4360
Serositis	15 (13.64)	03 (8.57)	0.5632
Renal disorders	**63 (57.27)**	**07 (20)**	**0.0002**
Neurological disorders	**14 (12.72)**	**00**	**—**
Cutaneous involvement	18 (16.36)	05 (14.29)	1.000
Hematological disorders	21 (19.09)	12 (34.29)	0.0687

**Table 3:** A meta-analysis of correlation between proinflammatory cytokines (IL-6, TNF-*α*, and IL-1*β*) and disease activity.

Population (number of patients)	Cytokine	Patients	Controls	Disease activity		Correlation with SLEDAI	Reference
				Active	Inactive		
Indian^a^ (145)	IL-6	63.00 ± 67.28	14.13 ± 8.61	70.45 ± 68.32	43.83 ± 63.36	*r* = 0.20, *P* = 0.01	Present study
	TNF-*α*	40.17 ± 40.46	17.35 ± 9.32	44.76 ± 68.32	25.97 ± 22.03	*r* = 0.27, *P* = 0.01	
	IL-1*β*	11.48 ± 9.97	7.89 ± 3.65	13.21 ± 10.76	6.23 ± 3.27	*r* = 0.38, *P* = 0.001	

Caucasian^b^ (40)	TNF-*α*	12.63 (7.53–21.04)	2.27 (1.77–3.18)	—	—	*r* = 0.330, *P* < 0.05	[22]
	IL-1*β*	2.8 (0.7–2.03)	0.98 (0.72–1.49)	—	—	*r* = 0.394, *P* < 0.001	

Brazilian^c^ (60)	TNF-*α*	2.18 (0.18–11.17)	1.30 (0.25–12.53)	—	—	*r* = 0.39, *P* = 0.002	[23]
	IL-6	1.5 (0.22–13.98)	0.98 (0.39–13.29)	—	—	No correlation	

Korean^d^ (166)	IL-6	0 (0, 3.8)	0 (0, 0)	3.3 (0, 12.2)	0 (0, 2.7)	*r* = 0.232, *P* = 0.01	[24]

Egyptian^a^ (40)	TNF-*α*	—	—	766.95 ± 357.82	314.01 ± 100.87	*r* = 0.743	[7]
	IL-6	—	—	135.4 ± 54.23	47.33 ± 18.61	*r* = 0.772	

Multiethnic^e^ (171)	IL-6	1.64 (0.05)	1.03 (0.03)	—	—	—	[25]

Swedish^f^ (52)	IL-6	23.7 (<13.5–156)	<13.5 (<13.5–29)	—	—	—	[26]

*r*: correlation coefficient. ^a^Mean ± SD, *P* value <0.05 (unpaired *t*-test). ^b^Median (interquartiles Q1–Q3), *P* value = 0.001 (Mann-Whitney *U* test). ^c^Median (range), *P* value <0.01. ^d^Median (25th percentile, 75th percentile), *P* value <0.001. ^e^White, Afro-Caribbean, South Asian, Chinese, and others, mean (standard error in the mean level), *P* value <0.01. ^f^IL-6 levels in sera measured by bioassay, median (range), *P* value <0.0005.
